# Evaluation of Therapeutic Effects of Computed Tomography Imaging Classification Algorithm-Based Transcatheter Arterial Chemoembolization on Primary Hepatocellular Carcinoma

**DOI:** 10.1155/2022/5639820

**Published:** 2022-04-22

**Authors:** Qiang Li, Guang Luo, Jian Li

**Affiliations:** ^1^Department of Medical Imaging, Heping Hospital Affiliated to Changzhi Medical College, Changzhi 046000, Shaanxi, China; ^2^Department of Radiology, Qingdao No. 6 People's Hospital, Qingdao 266033, Shandong, China; ^3^Department of General Surgery, Baoji Central Hospital, Baoji 721000, Shaanxi, China

## Abstract

To investigate the evaluation of therapeutic effects of computerized tomography (CT) imaging machine learning classification algorithm-based transcatheter arterial chemoembolization (TACE) on primary hepatocellular carcinoma (PHC), machine learning algorithm was optimized to propose the feature extraction of soft margin, analyze CT images, and acquire relevant texture features to assess if it can predict the multistage features of PHC for the application of the therapeutic effects of TACE on PHC. Besides, PHC patients receiving surgical excision were retrospectively collected, and then 483 patients with hepatocellular carcinoma (HCC) were determined from cases. After that, a total of 162 cases meeting the standards were selected. Besides, the features of images were classified and analyzed by machine learning algorithm, and volume of interest (VOI) images of patients in each group were acquired by image segmentation layer by layer. In addition, the texture features of images were extracted. The results showed that 5 CT image-based texture features, including 2 histogram features and 3 matrix-based features, all described the specificity and heterogeneity of tumors. The analysis of the diagnostic effectiveness of the evaluation of response group by each texture parameter demonstrated that its sensitivity, specificity, and area under curve (AUC) were 83.63%, 90.91%, and 0.08%, respectively. Based on CT prediction, machine learning algorithm was fused to realize excellent classification effects on multistage and multiphase features and offer imaging support to the clinical selection of reasonable therapeutic plans. In addition, multiphase and multifeature-based medical tumor classification method was put forward.

## 1. Introduction

Primary hepatocellular carcinoma (PHC) is one of the commonest cancers around the world [1]. There are about 650,000 new hepatocellular carcinoma (HCC) cases every year, and over half of the new cases are in China. HCC becomes one of the most hazardous malignant tumors [2]. At early phase of HCC, patients' clinical symptoms are not obvious and more recessive and undetectable compared with other cancers [3]. Besides, tumor metastasis and recurrence usually occur [4]. As a result, patients often miss the optimal treatment period. Even if they receive surgical treatment, 5-year survival rate reaches only 7% [5]. There are many local therapeutic methods for unresectable tumors, and the primary one is transcatheter arterial chemoembolization (TACE) [6]. Imaging examination is a significant means of diagnosing liver tumors, which can reflect the overall condition of tumors noninvasively. Therefore, medical imaging plays a more and more important role in early disease detection, diagnosis, and treatment. Radiomics mainly includes ultrasonography, computerized tomography (CT), positron emission tomography (PET)/CT, and magnetic resonance imaging (MRI). CT imaging is based mainly on the differences in density among different tissues, and it can provide more rich structural information for people [7].

The electronization and intelligence of medical imaging are gradually realized. Researchers attempt to adopt computers to help doctors interpret images [8]. However, medical image analysis task is different from natural image analysis task in computer vision field. With strong medical professionalism and high level of division, the number of images is often insufficient. What's more, annotation is expensive. Tagging sufficiently large datasets is usually time-consuming. The results of detection revealed highly imbalanced imaging data [9]. The number of normal images is much greater than that of abnormal images. The key cause is poor interpretability of deep network, which is the focus adopted by doctors and patients. Therefore, whether deep network is the preferred choice for any task and the circumstance under which other methods can be adopted to achieve objectives simply and efficiently are significant problems worthy to be studied in medical field. In computer classification algorithms, machine learning is an algorithm that is complex mode in a type of learning experience data and makes accurate predictions. It is divided into supervised learning, semisupervised learning, and unsupervised learning [10]. Lv et al. [11] used CT images of machine learning algorithm for clinical analysis of liver cancer treatment and achieved good prediction and evaluation.

Based on different detection and imaging principles, richer and richer imaging modes emerge and can accurately describe the different conditions of objects being measured [12]. The analysis of the preoperative images of PHC patients treated with TACE by computer classification algorithms was investigated, and radiomics, image features, and clinical data were fully utilized to establish more precise prediction models to provide the direction for accurate treatment of HCC. In addition, the correlation of the changes among the extracted features based on machine learning algorithm, the early, middle, and late phases of innovative mining artery, and liver and gallbladder phase, and the classification effectiveness of multiphase information feature fusion were discussed.

## 2. Materials and Methods

### 2.1. Subjects

The patients with PHC undergoing surgical excision at hospital between January 2018 and January 2021 were collected retrospectively. After that, a total of 483 patients were determined from electronic medical records. There were total 162 cases conforming to inclusion and exclusion standards. All patients volunteered to participate in the project and had signed the informed consent forms before the implementation of the project. This study has been approved by the ethics committee of the hospital and all patients had signed the informed consent.

Inclusion criteria:

1. All patients were diagnosed with PHC
2. All patients received complete baseline enhancement CT examination 1 week before surgery
3. All patients did not receive any drug and relevant surgical treatment before baseline examination
4. All patients received appropriate iodized oil plans for TACE treatment after baseline examination
5. The examination of therapeutic effects was implemented 6 months after the 1^st^ TACE surgery

Exclusion criteria:

1. Patients ever received local tumor treatment, such as cryotherapy and radiotherapy
2. Patients suffered from nonhepatocellular PHC validated by other pathological symptoms
3. Over two tumors existed in patients' livers

### 2.2. Examination Methods

CT examination was carried out. Patients were forbidden to eat 8 hours before the examination and asked to take supine position. The scan parameter was as follows. Layer thickness was 5 mm, layer spacing was 5 mm, tube voltage was 120 kV, and exposure time was 0.5 s. Iopromide was utilized as contrast medium. The drug was administered through cubital vein with 2.0–2.5 mL/s. After the injection of contrast medium, arterial phase, portal venous phase, and lag phase scans were performed. The scan covered all lesion areas [13].

### 2.3. Machine Learning Algorithm

**Figure 1:**
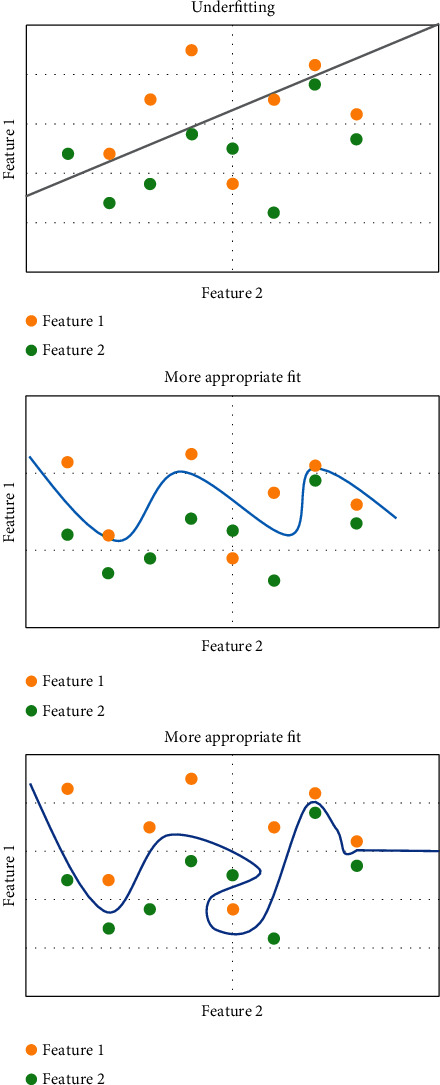
Underfitting, appropriate fitting, and overfitting.

In supervised learning, overfitting is the biggest obstacle in the training process. Overfitting refers to the excessive presentation of the features of training sets by learning algorithm, which shows excellent effects on training sets but poor effects on test sets [14]. Underfitting refers to the inadequate training of algorithm without realizing excellent effects on training sets.  Figure 1 shows underfitting, appropriate fitting methods, and overfitting scenes as follows.

Support vector machine (SVM) is a dichotomic classification model, which is aimed at finding a hyperplane to divide data into two types and then to maximize the margins between the hyperplane and two types of data feature points.

Mathematical modeling was performed for the above problems, and the process of solving hyperplane was to solve the constrained minimization problem in the following equation:

(1) $$\begin{array}{c} \underset{N,a}{\min}\frac{1}{2}{\left|\left|N\right|\right|}^{2}\;\\ \text{s.t. }\;y_{f}\left(N^{T}x_{r}+a\right)\geq 1. \end{array}$$

In equation (1), *r* = 1,2,...,*m*, *y*_*f*_ denoted sample labels, *x*_*r*_ referred to sample features, *N* represented the weight of features, and *m* meant the number of features.

In the above case, the assumption that the trained samples were linearly separable needed to be realized. In terms of linear inseparability, data needed to be mapped to high-dimension space to transform the problem into linear separability. In this case, the mathematical model of SVM was expressed by the following equation:

(2) $$\begin{array}{c} \underset{N,a}{\min}\frac{1}{2}{\left|\left|N\right|\right|}^{2}\;\\ \text{s,t.}\;y_{f}\left(N^{T}\emptyset \left(x_{r}\right)+a\right)\geq 1,r=1,2,\ldots \;,m. \end{array}$$

In equation (2), ∅(*x*_*r*_) referred to the features of samples after *x*_*r*_ being mapped.

Lagrange multiplier method was utilized to acquire the dual issue of the problem, which facilitated further solution of the mathematical model. The equation of the model is expressed by the following equation:

(3) $$\begin{array}{c} \underset{\beta }{\max}\overset{m}{\underset{r=1}{\sum }}\beta_{r}-\frac{1}{2}\overset{m}{\underset{i=1}{\sum }}\overset{m}{\underset{l=1}{\sum }}\beta_{i}\beta_{l}y_{i}y_{l}\emptyset {\left(x_{i}\right)}^{T}\emptyset \left(x_{l}\right)\\ \text{s,t. }\overset{m}{\underset{r=1}{\sum }}\beta_{r}\beta_{l}=0,\;\beta_{r}\geq 0,r=1,2,\ldots ,m. \end{array}$$

Β_*r*_ represented Lagrange multiplier. In the above equation, the inner product after the mapping of samples to high-dimension space ⊘(*x*_*i*_)^*T*^⊘(*x*_*l*_) needed to be calculated. Because of its high dimension, which was even infinite, the calculation was very difficult. Therefore, kernel function was introduced, as the following equation:.

(4) $$\begin{array}{c} W\left(x_{r},x_{l}\right)=\emptyset {\left(x_{r}\right)}^{T}\emptyset \left(x_{l}\right). \end{array}$$

**Figure 2:**
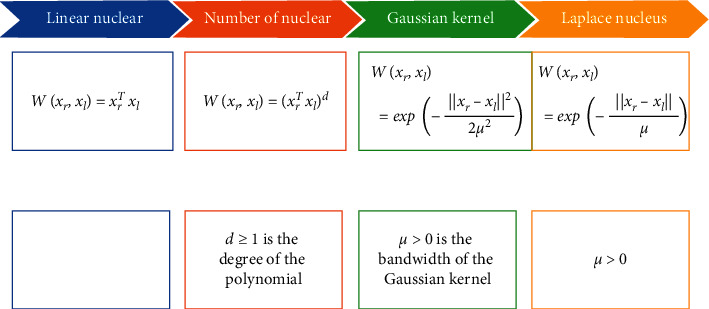
Several common kernel function types.

By the direct substitution of low dimension features into the kernel function, the high-dimension transformation of the features was acquired equivalently, and then the inner product of the transformed features was solved.  Figure 2 displayed different feature mapping methods corresponding to different kernel functions as follows [15].

In the previous introduction, it was required that all samples were divided correctly, which was called hard margin. However, there might be some noisy points in data. As a result, the utilization of kernel function still could not achieve linear separability. In some cases, separability was realized, but the selection of hyperplane might be greatly affected [16]. Hence, SVM was allowed to make mistakes on some samples, and soft margin was introduced.

Its mathematical model was modified as shown in the following equation:

(5) $$\begin{array}{c} \;\underset{N,a}{\min}\frac{1}{2}{\left|\left|N\right|\right|}^{2}+C\overset{m}{\underset{r=1}{\sum }}\theta_{r}\\ \text{s,t. }y_{f}\left(N^{T}x_{r}+a\right)\geq 1-\theta_{r},\;\theta_{r}\geq 0,\;r=1,2,\ldots ,m. \end{array}$$

In equation (5), *θ*_*r*_ is the slack variable, which represented the degree of inaccurate sample classification. After the mathematical model was solved, the corresponding hyperplane was acquired.

### 2.4. Image Collection and Feature Analysis

**Figure 3:**
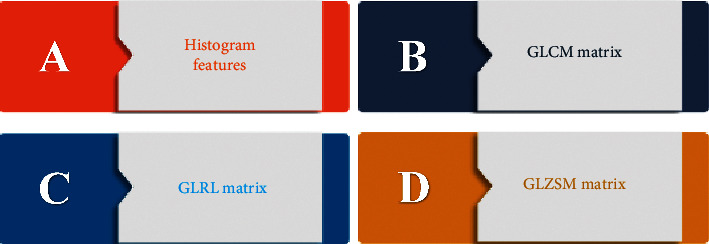
Classification of types of texture features.

CT scan images were analyzed by picture archiving and communication systems (PACS) and then loaded into the system. Besides, ITK-SNAP was adopted to sketch volume of interest (VOI) in liver CT images and portal vein (PV) enhanced images of patients layer by layer, as demonstrated in Figure 3. VOI sketch was completed by a radiologist with 10-year work experience and then reviewed by another senior radiologist. The selection of lesions of interest was targeted at the lesions whose diameter was greater than 1 cm. The sketch was implemented by delineating along the edge contours of lesions and completely covering whole tumors. During the sketch, the radiologist needed to avoid large vessels. The features of the sketched VOI images were extracted and analyzed which was based on medical imaging toolkit (MITK) platform.

### 2.5. Image Features

Based on previous studies, the following features were summarized [17]:

- In terms of tumor edge, tumors had clear boundaries at arterial phase or PV phase. Envelopes could be observed, and overall tumor nodules were closed [18]. On the contrary, tumor edge was not obvious, and peripheral multiple nodules or invasive growth appeared. Tumor edge was in the form of burr without envelopes.
- In terms of peritumoral enhancement, enhancement lesions appeared around tumors at arterial phase. Compared with the background of livers, enhancement lesions showed a declining trend in CT images at equilibrium phase.
- In terms of halo signs, low-density rings appeared around tumors, and the periphery of tumors was partially or completely surrounded at PV phase.
- In terms of arteries inside tumors, scattered arterial vessel enhancement persisted in tumors at arterial phase [19].
- In terms of tumor-liver differences, the differences between liver parenchyma density and tumor density were significant, and the former one was higher than the latter one in CT images during arterial phase [20].

### 2.6. Extraction of Texture Features

The types of texture features to be acquired included histogram features and three matrix-based features, including gray-level co-occurrence matrix (GLCM) feature, gray-level run length matrix (GLRLM) feature, and gray-level zone size matrix (GLZSM) feature. Figure 3 shows the specific classification of features as follows.

### 2.7. Extraction and Construction of Multiphase Features

The independently developed feature extraction platform was utilized to extract the total volume features of the early, middle, and late phases of arteries as well as HCC at liver and gallbladder phase, including 12 first-order statistical features, 96 texture features (four directions included 0′, 45′, 90′, and 135′), and 7 morphological features. The average values of the four directional features were utilized as the final value of the texture feature 89. As a result, the number of features at each phase was 12 + 24+7 = 43. The total number of features at four phases was (12 + 24)*∗*4 + 7 = 115.

In addition, it was found out that the images formed at the arterial early, middle, and late phases of HCC and liver and gallbladder phase showed the representation of HCC of the same patient at different time. The variations among features might also contain information. Therefore, the features of change rate of first-order statistical features and texture features between two adjacent phases were constructed based on the original features. The construction method was shown in the following equation:

(6) $$\begin{array}{c} {\tilde{x}}_{l-r}=\frac{x_{l}-x_{r}}{x_{r}}. \end{array}$$

In equation (6), *x*_*l*_ represented the feature *x* on phase *l*, and *x*(∼)_*l*−*r*_ referred to the change rate of feature *x* between phases *l* and *r*.

### 2.8. Model Assessment Methods

The generalization performance of machine learning algorithm was usually tested by experiment. To be specific, the sample was divided into training set and test set. The model was trained on training set. On the test set, the discriminant ability of the model to new samples was tested and adopted as the approximation of model generalization performance. The main ways to divide training set and test set included hold out method, cross validation method, and bootstrapping method.

### 2.9. Evaluation of Therapeutic Effects

Therapeutic effects were assessed by response evaluation criteria in solid tumors (RECIST). CT patients were divided into two groups, including ease group and relief group. Ease group included complete response (CR) patients and partial response (PR) patients. Relief group included progressive disease patients (PD) and stable disease (SD) patients [21].

### 2.10. Statistical Analysis

Statistical product and service solutions software was utilized for statistical analysis. The texture parameters that showed statistical meaning and could effectively distinguish patients in different groups were performed with receiver operating characteristic (ROC) analysis. Besides, the corresponding areas under curve (AUC), sensitivity, and specificity were calculated. AUC values greater than 0.9 indicated excellent prediction effects, AUC ranging between 0.7 and 0.9 revealed good prediction effects, and AUC values less than 0.7 demonstrated average prediction effects. *P* < 0.05 meant that the differences showed statistical meaning.

## 3. Results and Discussion

### 3.1. General Features of Subjects

**Figure 4:**
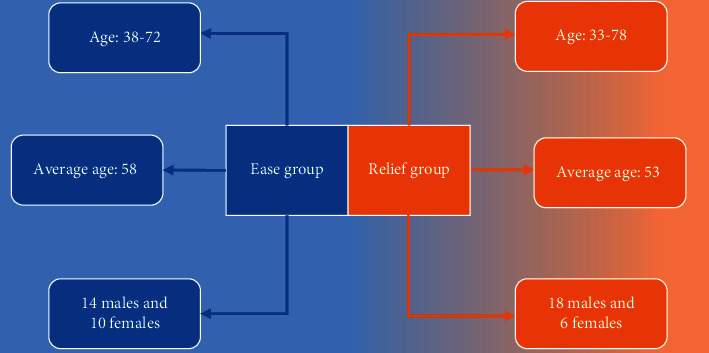
Details of patients in the ease group and relief group.

According to the inclusion and exclusion standards, there were total 48 patients meeting the requirements. Based on RECIST criteria, patients were divided into ease group (24 cases in 1 group) and relief group (24 cases in 2 groups) 6 months after TACE treatment based on therapeutic effects, as Figure 4 illustrates.

### 3.2. Machine Learning Algorithm-Based Morphological Features

**Figure 5:**
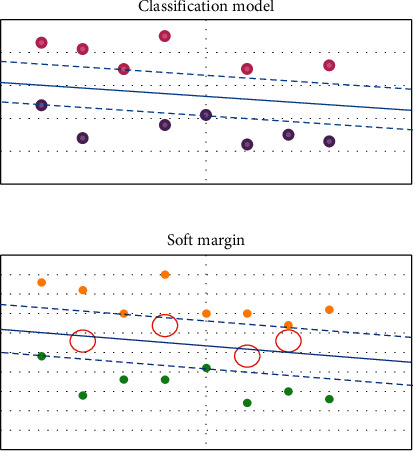
Data features before and after division of types of soft margins.

Based on machine learning algorithm, the morphological features that could significantly judge if there was microvascular invasion (MVI) in HCC included volume, surface area, density, maximum diameter, and sphericity. After the removal of relevant features, the remaining morphological features included sphericity, volume, surface area, and density (Figure 5).

**Figure 6:**
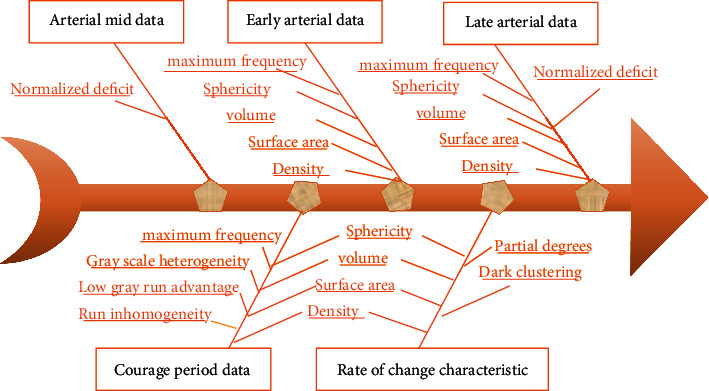
Specific details of 5 feature datasets.

In terms of the features of change rate, the change rate of skewness during arterial middle-late phases, the change rate of cluster-shade during arterial middle-late phases, and the change rate of run inhomogeneity during arterial late phase and liver and gallbladder phase could significantly judge if there was MVI in HCC, and the linear correlation among the above change rates was low. The above three types of features were synthesized to establish 5 feature sets at last.  Figure 6 shows the features included in each data set as follows.

**Figure 7:**
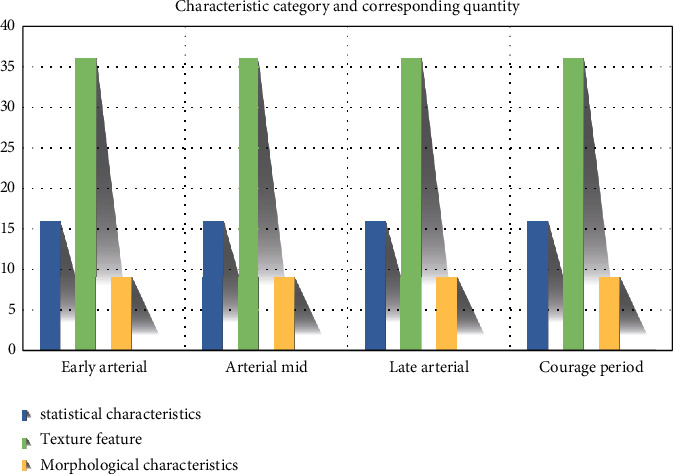
Summary of multiphase feature quantity.

The images formed at the arterial early, middle, and late phases of HCC as well as liver and gallbladder phase showed the representation of HCC of the same patient at different time. Based on the original features, the features of the change rates of first-order statistical features and texture features between two adjacent phases were constructed (Figure 7).

### 3.3. Evaluation of Therapeutic Effects

**Figure 8:**
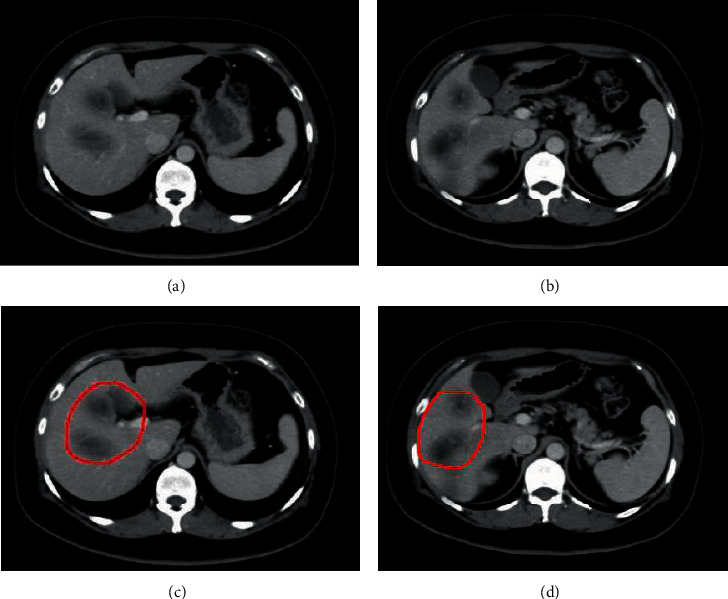
Preoperative CT images of a PHC patient and ROI sketch.

Based on machine learning algorithm classification, the preoperative images of PHC patients were sketched and then included in the scope of the evaluation of therapeutic effects. Besides, the ROI sketch was assessed based on feature points (Figure 8).

**Figure 9:**
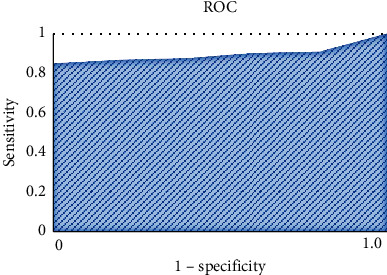
AUC of texture features.

Correlation test method was utilized to reduce and remove the redundancy among each radiomics feature. Based on the grouping of CT patients (group 1 and group 2), patients in different groups received *t* test to acquire 42 texture features, including histogram feature, GLCM feature, GLRLM feature, and GLZSM feature. Besides, least absolute shrinkage and selection operator (LASSO) was adopted in dimensionality reduction and modeling to acquire 5 meaningful texture features that could distinguish patients in different groups, including 2 histogram features (kurtosis and histogram energy), 2 GLZSM features (high-intensity emphasis (HIE) and high-intensity large area emphasis (HILAE)), and 1 GLCM feature (sum entropy). In addition, ROC curves (Figure 9) were adopted to analyze the diagnostic effectiveness of the evaluation of response group by each texture parameter, and the results demonstrated that its sensitivity, specificity, and AUC were 83.63%, 90.91%, and 90.08%, respectively.

## 4. Discussion

HCC is a malignant tumor with high heterogeneity, which is featured with insidious onset without obvious symptoms in most cases. At the time of diagnosis, most patients suffered from HCC at the middle and late phases. HCC progresses rapidly with poor therapeutic effects and prognosis [22]. For those patients with poor prognosis, the follow-up intervals can be shortened appropriately and proper prevention measures can be taken, such as postoperative TACE treatment or sorafenib auxiliary treatment [23]. Patients with relatively better prognosis are exempt from excessive treatment. Besides, auxiliary therapies can be reduced and even replaced by long-term follow-up visits. With the great development of computer programming technology and hardware in recent years, imaging processing is continuously updated. The application of medical images becomes a hot topic. At present, it is gradually possible to realize the standardization of medical imaging processing by different devices. With the help of engineering algorithms, high-throughput extraction was performed on the intensity, texture, shape, wavelet transform, and other quantitative features of CT images for further quantitative analysis of lesion areas of the whole CT images. Finally, rich and accurate information was acquired.

The texture features of preoperative enhanced CT images of PHC patients receiving TACE treatment were extracted to establish the corresponding preoperative therapeutic effect prediction model. The results demonstrated that the sensitivity, specificity, and AUC of CT-based radiomics model were 83.63%, 90.91%, and 90.08%, which indicated good prediction effectiveness. Radiomics features were closely correlated with the microstructure of tumors and biological behaviors. In addition, there was specificity among different tumors of different individuals, which resulted in the specificity of their radiomics features. A total of 5 CT image-based texture features were discovered, including 2 histogram features and 3 matrix-based features. Histogram was first-order statistics, which was mainly adopted to describe the intensity or brightness information in tumors [24]. In contrast, matrix-based features were second-order statistics that could be utilized to describe the complexity, changes of layer structure, and texture thickness in tumors [25]. The above feature information described the specificity and heterogeneity of tumors together. What's more, the best boundary of multiphase high-dimension features was found by machine learning algorithm for MVI classification of HCC. The features of the change rates in HCC dynamic enhanced images at the arterial early, middle, and late phases as well as liver and gallbladder phase between two adjacent phases were innovatively proposed and developed. Compared with the feature sets directly acquired from the four datasets, including the arterial early, middle, late, and liver and gallbladder phases, the model acquired based on the training of change rate feature sets obtained the best classification effectiveness. AUC value and accuracy on training sets were both higher than 0.7. Because most image data of research institutes and medical organizations in different regions could not be shared, the established database might show local limitations without sufficient representativeness. Hence, the current study and application of radiomics show a large number of limitations. The objective conditions limit the research progress of radiomics to some degree and affect the unification of the results of radiomics and clinical application.

## 5. Conclusion

The project focused on the classification algorithm of medical tumor images. Besides, the assessment of the therapeutic effects of TACE treatment on PHC and the classification of features were implemented.

The innovations of the project were as follows.

- )Computer network was applied in the classification task of HCC microvascular features. In terms of sensitivity index, a two-fold performance improvement was achieved.
- The change rate features among HCC multiple phases were firstly proposed and applied in HCC multiphase classification. The classification effects were superior to existing indexes.

The main achievements were as follows:

- )Enhanced CT images were analyzed to acquire the corresponding texture features and construct CT-based radiomics model. The model could effectively predict the postoperative effects of TACE treatment on PHC patients. Besides, machine learning algorithm was fused based on CT prediction to realize good classification effects on multiphase and multiterm features, which provided imaging support for the clinical selection of reasonable therapeutic plans.
- Multiphase and multifeature-based medical tumor imaging classification method was proposed. The independently developed multimode medical image feature extraction platform was utilized to extract the total volume first-order statistical features, morphological features, and texture features of arterial 3 phases in HCC dynamic enhanced MRI. Besides, the imbalanced data were processed and the change rate features among HCC multiple phases were innovatively proposed and developed. What's more, the feature sets fusing multiple phases were established, machine learning algorithm was trained, and the classification of HCC MVI was realized. Compared with that of single feature set and single-phase feature set, the classification performance of the included feature sets was further enhanced.

However, there are several limitations in the project. Firstly, the sample size is small compared with numerous variables. Therefore, the subsequent implementation of large-scale clinical studies to enlarge sample size promotes the validation and improvement of the applicability of models. Based on the validated and improved applicability of models, multimode and multiphase deep network framework can be constructed. Besides, the effectiveness of data-driven learning fusing multiple modes and multiple phases needs to be further explored and the definition of the features learned by the network should also be investigated. Secondly, the limitation lies in therapeutic effects. Single TACE treatment of PHC can still be greatly improved. Therefore, the therapeutic effects of TACE combined with other therapies in subsequent clinical treatment will be further improved.

## Data Availability

The data used to support the findings of this study are available from the corresponding author upon request.
